# Age-specific disparity in insomnia among COVID-19 patients in Fangcang shelter hospitals: a population-based study in Shanghai, China

**DOI:** 10.3389/fneur.2024.1420898

**Published:** 2024-07-26

**Authors:** Ruizi Shi, Yihui Wang, Ying Chen, Zhitao Yang, Feng Jing, Hanbing Shang, Erzhen Chen, Ying Zhou

**Affiliations:** ^1^Shanghai Institute of Aviation Medicine, Ruijin Hospital, Shanghai Jiao Tong University School of Medicine, Shanghai, China; ^2^Department of Emergency, Ruijin Hospital, Shanghai Jiao Tong University School of Medicine, Shanghai, China; ^3^Division of Medical Affairs, Ruijin Hospital, Shanghai Jiao Tong University School of Medicine, Shanghai, China; ^4^Nursing Department, Ruijin Hospital, Shanghai Jiao Tong University School of Medicine, Shanghai, China; ^5^Department of Neurosurgery, Ruijin Hospital, Shanghai Jiao Tong University School of Medicine, Shanghai, China; ^6^Department of Neurosurgery, Ruijin-Hainan Hospital, Shanghai Jiao Tong University School of Medicine, Haikou, China

**Keywords:** insomnia, Fangcang shelter hospital, SARS-CoV-2, age differences, SARS coronavirus, vaccine

## Abstract

**Background:**

Fangcang shelter hospitals are quarantine facilities offering primary medical treatment for mild and asymptomatic SARS-CoV-2 cases. Little is known about the age-specific prevalence of insomnia among patients in Fangcang shelter hospitals, particularly in older age groups.

**Methods:**

This cross-sectional study was conducted in the three largest Fangcang shelter hospitals during the lockdown period, from March to May 2022, in Shanghai. The patients’ demographic and medical information was recorded. Insomnia was defined according to the prescriptions for zolpidem and estazolam. The overall and age-specific prevalence and the risk factors of insomnia were investigated through regression models.

**Results:**

A total of 2,39,448 patients were included in this study (59.09% of the patients were male, the median age was 42, and 73.41% of the patients were asymptomatic), with the prevalence of insomnia being 3.1%. The prevalence of insomnia varied across different age groups (<18 years: 0.23%, 18–64 years: 2.64%, and ≥65 years: 10.36%). SARS-CoV-2 vaccine, regardless of the number of doses, was significantly associated with a decreased risk of insomnia for the group aged ≥65 years. Three doses of the vaccine reduced the risk of insomnia for patients aged 18–64 years. An extra day in the hospital significantly increased the risk of insomnia by approximately 10% for all age groups. Mild symptoms were significantly associated with a higher risk of insomnia among patients aged <65 years old, while being male and residing in the surrounding area were negatively associated with insomnia for all adults.

**Conclusion:**

This study observed that older patients were a high-risk population for developing insomnia in Fangcang shelter hospitals. SARS-CoV-2 vaccination might decrease the risk of insomnia in adults, especially the older adult, which indicates the benefits of vaccination for reducing insomnia among infected patients.

## 1 Introduction

Since December 2019, the coronavirus disease 2019 (COVID-19), caused by severe acute respiratory syndrome coronavirus 2 (SARS-CoV-2), has been spreading throughout the world. Since traditional approaches were limited regarding curbing the disaster caused by this infectious disease, Fangcang shelter hospitals, characterized by their rapid construction, massive scale, and low building and operation costs that make them particularly well suited to address public health emergencies, were developed in many cities of China to admit massive numbers of patients for treatment (1, 2). Therefore, in late February 2022, when an outbreak of the Omicron wave of the COVID-19 pandemic hit Shanghai (3), numerous SARS-CoV-2-infected residents in Shanghai were isolated and treated in Fangcang shelter hospitals (4), which were once again improved to be a critical measure for patient care and pandemic control in the following months (5).

Several investigations have demonstrated a strong correlation between coronavirus and sleep disorders. A previous meta-analysis estimated and reported an increased prevalence of insomnia during and after the severe coronavirus outbreak in 2019 (6). Some studies used self-reported questionnaires to assess and report sleep problems during the COVID-19 pandemic (7, 8). More recently, a study examining the sleep quality of participants during the COVID-19 transmission wave since December 7, 2022, using the online questionnaire, the Pittsburgh Sleep Quality Index (PSQI), found that 36.8% of participants reported poor sleep quality (9). However, these studies did not focus on patients in Fangcang shelter hospitals. Several studies suggested that SARS-CoV-2-infected patients in Fangcang shelter hospitals were prone to sleep disorders due to continuous bright light even at night, the lack of personal space, environmental noises, attitude toward infection, and prognosis, as well as dysfunctional beliefs about sleep (10–13). Gu et al. (14) reported that 14.8% of healthcare workers experienced insomnia in a Jianghan Fangcang shelter hospital from February 21 to February 28, 2020. However, the sleep problems of healthcare workers might be different from the patients’ due to their role in Fangcang shelter hospitals. Moreover, different SARS-CoV-2 variants showed varied impacts on the severity of clinical symptoms, outcomes, and related health problems.

Numerous mutations in the spike protein of the Omicron variant increased its transmissibility and allowed for viral escape by evading the immune response. The variant generated false negative results in polymerase chain reaction assays due to “S gene target failure” (15, 16). One of the characteristics of the Omicron variant was that it caused quick spreading of COVID-19 infection with massively mild and asymptomatic carriers (17). A single-center cross-sectional study reported that 34.3% of asymptomatic COVID-19 carriers in a Fangcang shelter hospital were observed to have poor sleep quality. This was assessed using the PSQI from March 2022 to April 2022 (18). Another study reported that approximately 3.6% of patients admitted to Fangcang shelter hospitals in Shanghai used associated psychiatric drugs, and more than half of the patients used psychiatric drugs to treat insomnia (19). To the best of our knowledge, however, there has been limited research on the physiological effects of COVID-19 infection on insomnia across different age groups during the Omicron wave.

Different risk factors could affect the sleep health of people at different ages as sleep health varies widely across the lifespan. This was especially evident in Fangcang shelter hospitals during the late period of the COVID-19 pandemic. It is crucial to understand the sleep issues experienced by patients belonging to different age groups during the COVID-19 pandemic in Fangcang shelter hospitals for managing and meeting the needs of patients during any future outbreaks and for carrying out prompt interventions for vulnerable groups. We conducted a multicenter exploratory study to investigate the age-specific prevalence and risk factors of insomnia among COVID-19 patients during the Omicron wave in the three largest Fangcang shelter hospitals in Shanghai. The specific goals of the study included the following: (1) characterization of all asymptomatic and mild COVID-19 patients in Fangcang shelter hospitals; (2) identification of the age-specific prevalence of insomnia; and (3) investigation of the risk factors associated with insomnia among COVID-19 patients in Fangcang shelter hospitals.

## 2 Methods

### 2.1 Study design and enrollment

This multicenter, cross-sectional study was conducted in three of six provincial-level Fangcang shelter hospitals in Shanghai, China, including the National Exhibition and Convention Centre (NECC) in Qingpu district, the Shanghai New International Expo Centre (SNIEC) in Pudong district, and the World Expo Museum (WEM) in Huangpu district.

The National Exhibition and Convention Centre was the largest Fangcang shelter hospital, which received 1,69,922 infected patients from 26 March 2022 to 31 May 2022. The Shanghai New International Expo Centre was the first provincial-level Fangcang shelter hospital with a bed capacity of more than 10,000 beds. A total of 46,695 infected patients were admitted from 9 April 2022 to 3 May 2022. The World Expo Museum was the first huge Fangcang shelter hospital to complete the admission task, with a total of 25,647 SARS-CoV-2-infected patients cured from 26 March to 25 May 2022 (Supplementary Table S1).

All patients received physical evaluations, but only those who were diagnosed as asymptomatic or with mild symptoms were eventually admitted to Fangcang shelter hospitals. Fangcang shelter hospitals provide primary medical services, including health management, medical observation, and targeted treatment, combined with traditional Chinese medical care, to help patients recover and be discharged. The exclusion criteria included the following: patients who provided missing or obviously incorrect information and patients who had a self-reported history of psychiatric disorders. This study was approved by the Institutional Review Board (IRB) of Ruijin Hospital, affiliated with the Shanghai Jiao Tong University School of Medicine, and all methods were carried out in accordance with the approved guidelines.

### 2.2 Assessment of outcomes

Patients’ demographic and medical information was recorded in Fangcang shelter hospitals. We collected all information about the prescription medications of participants during their stay at the Fangcang shelter hospitals. All information about the psychiatric medications prescribed to COVID-19 patients is provided in a list in Supplementary Table S2. In this study, we followed the approach of a previous study where insomnia was defined as a condition that required a prescription for zolpidem and estazolam in consideration of their clinical application (19). Zolpidem is a short-acting non-benzodiazepine sleeping pill that is frequently used as the first line of treatment for individuals with episodic or incipient insomnia. Estazolam is a long-acting benzodiazepine sleeping drug, also known as sulazepam, with a variety of effects. Thus, it is frequently used in the treatment of moderate, severe, and chronic insomnia, and it can also treat sleep disturbances induced by aberrant emotions such as worry, despair, and stress. It is not feasible to diagnose insomnia in a timely manner using ICSD-D and DSM-5 criteria or using any other sleep problem scales because of the limited number of healthcare workers and the large number of patients to be treated.

### 2.3 Measurement and data collection

Demographic data were recorded in the information system of Fangcang shelter hospitals, namely age (0–17, 18–64, and ≥65 years), sex (male or female), marital status (single, married, and others), place of residence (central districts and surrounding areas), doses of vaccine (none, one or two, and more than two), discharge diagnosis (asymptomatic and mild symptoms), and the length of hospital stay. Central districts in Shanghai included the Huangpu district, Xuhui district, Changning district, Yangpu district, Jing’an district, Hongkou district, and Putuo district. Other districts, besides central districts, were categorized as surrounding areas. Asymptomatic and mild infection of SARS-CoV-2 was defined according to the Chinese Diagnosis and Treatment Protocol for Novel Coronavirus Pneumonia (Version 9). The category of mild infections included patients without dyspnea and with a decrease in oxygen saturation, progressive symptoms, pneumonia signs in chest imaging, or the need for escalation of medical care. Patients without any symptoms were defined as asymptomatic. The length of hospital stay was defined as the date of discharge minus the date of admission to Fangcang shelter hospitals.

### 2.4 Statistical analysis

Data analysis was performed using Stata 15.0. Continuous data were presented as mean (± standard deviation, SD) or median (interquartile range, IQR); group comparisons were assessed by conducting student’s *t*-test and the Kruskal–Wallis test, as appropriate. Categorical data were tabulated as numbers and percentages and assessed by conducting the chi-squared (*χ*^2^) test. The overall and age-specific prevalence of insomnia was calculated. We performed multivariate regression analysis to calculate the adjusted odds ratio (aOR) of correlates associated with insomnia. We constructed the interaction between age and all other correlates to see if there was any age-specific effect of the correlates on insomnia. We then put each interaction term into regression models individually to obtain the *p*-value for each interaction. There was only one interaction per model. In addition to age, potential explanatory variables that were examined in the multivariate regression model included sex, vaccination status, marital status, being diagnosed with a mild infection, the location of shelter hospitals, case resource, and the length of hospital stay. Therefore, we ran models that included all participants and were stratified by three age groups. In the three age-specific models, age was also included as a continuous variable. A *p*-value of <0.05 was considered statistically significant.

## 3 Results

### 3.1 Characteristics of participants

**Figure 1:**
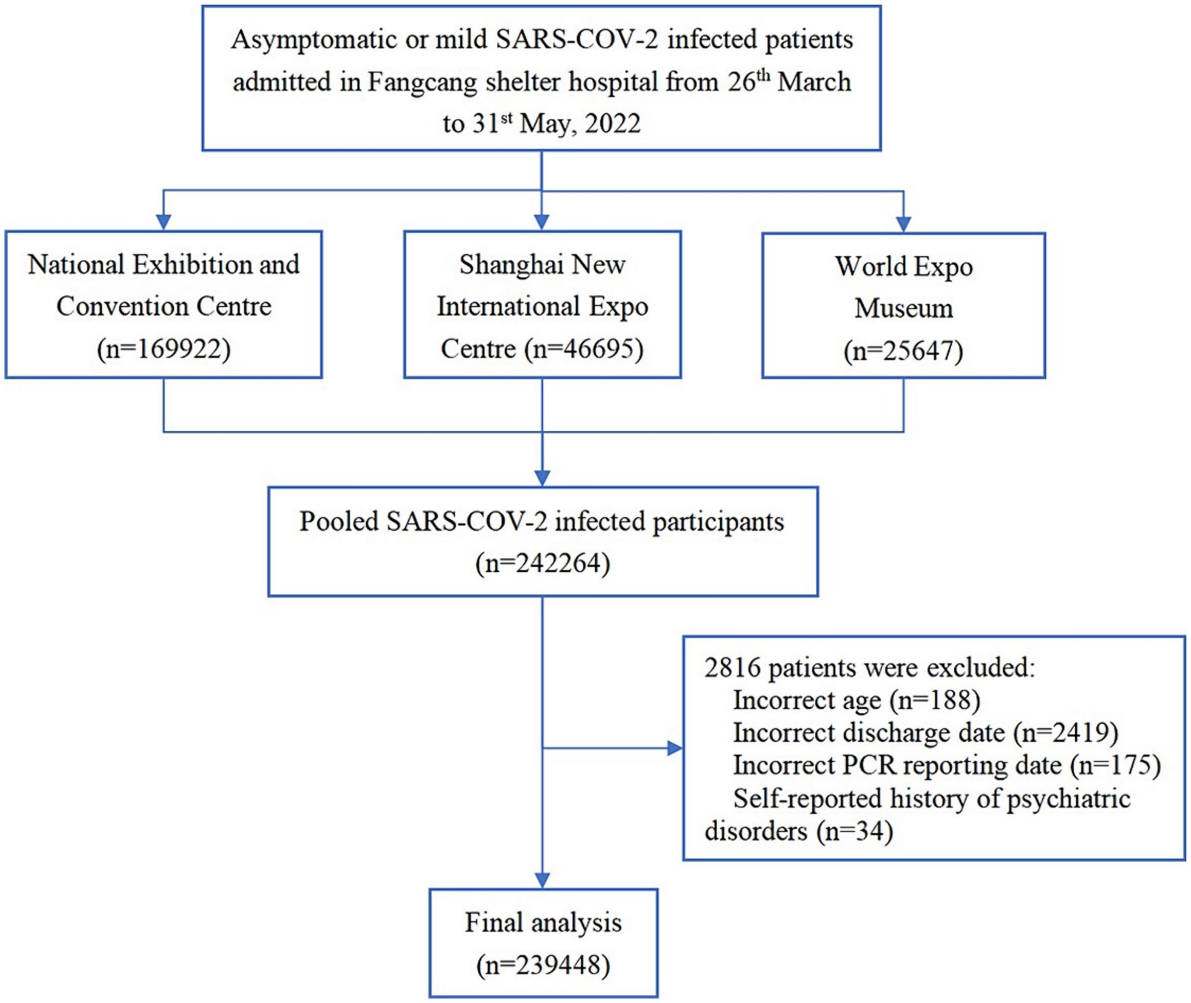
Flowchart of the sample selection process.

A total of 2,42,264 patients were hospitalized in the three largest Fangcang shelter hospitals between 26 March 2022 and 31 May 2022. According to the inclusion and exclusion criteria, a total of 2,39,448 cases were included in the final analysis after 2,816 patients with missing or incorrect information were excluded (Figure 1). Table 1 summarizes the demographic characteristics of the study population. The median age of the participants was 42 (IQR:30–54) years, with a higher proportion of male-to-female participants (59.09% vs. 40.91%). A total of 52.63% of SARS-CoV-2 infected patients were from the surrounding areas of Shanghai, 73.41% were asymptomatic, and 42.68% had three doses of the vaccine. The median time of the length of hospital stay was 7.0 (IQR: 5.0–10.0) days. People aged <18, 18–64, and ≥65 years made up 4.72, 87.41, and 7.87% of the participants, respectively.

**Table 1:** Characteristics of COVID-19 patients in Fangcang shelter hospitals.

Variable	Overall, *N* (%)	Age groups			*p*-value
		<18 years *N* (%)	18–64 years *N* (%)	≥65 years *N* (%)	
Total, *n*	239,482 (100)	11,305 (100)	209,328 (100)	18,849 (100)	
Age, median (IQR)	42 (30–54)	10 (7–14)	41 (31–52)	68 (66–70)	**<0.001**
Sex					**<0.001**
Female	97,956 (40.91)	4,912 (43.45)	84,144 (40.20)	8,900 (47.23)	
Male	141,492 (59.09)	6,393 (56.55)	125,155 (59.80)	9,944 (52.77)	
Marital status					**<0.001**
Unmarried	88,154 (36.82)	11,102 (98.20)	74,135 (35.42)	2,917 (15.48)	
Married	143,409 (59.89)	0 (0.00)	128,616 (61.45)	14,793 (78.50)	
Others	7,885 (3.29)	203 (1.80)	6,548 (3.13)	1,134 (6.02)	
Place of residence					**<0.001**
Central districts	113,434 (47.37)	5,773 (51.07)	96,001 (45.87)	11,660 (61.88)	
Surrounding areas	126,014 (52.63)	5,532 (48.93)	113,298 (54.13)	7,184 (38.12)	
Number of vaccine doses					**<0.001**
None	54,169 (22.95)	4,369 (38.76)	42,197 (20.48)	7,603 (40.68)	
One or two	81,122 (34.37)	6,697 (59.41)	69,505 (33.73)	4,920 (26.32)	
Three	100,748 (42.68)	207 (1.84)	94,372 (45.8)	6,169 (33)	
Discharge diagnosis					**<0.001**
Mild	175,778 (73.41)	8,898 (78.71)	153,267 (73.23)	13,613 (72.24)	
Asymptomatic	63,670 (26.59)	2,407 (21.29)	56,032 (26.77)	5,231 (27.76)	
Location of Fangcang shelter hospitals					**<0.001**
NECC	167,367 (69.90)	7,100 (62.80)	146,718 (70.10)	13,549 (71.90)	
WEM	25,593 (10.69)	1,122 (9.92)	23,131 (11.05)	1,340 (7.11)	
SNIEC	46,488 (19.41)	3,083 (27.27)	39,450 (18.85)	3,955 (20.99)	
Length of hospital stay, median (IQR)	7.0 (5.0–10.0)	7.0 (5.0–9.7)	7.0 (5.0–10.0)	8.0 (5.5–11.0)	**<0.001**

Central districts in Shanghai included the Huangpu district, Xuhui district, Changning district, Yangpu district, Jing’an district, Hongkou district, and Putuo district. Surrounding areas indicated other districts excluding central districts. NECC, National Exhibition and Convention Center; WEM, World Expo Museum; SNIEC, Shanghai New International Expo Centre. Texts in bold type indicate statistical significance.

**Figure 2:**
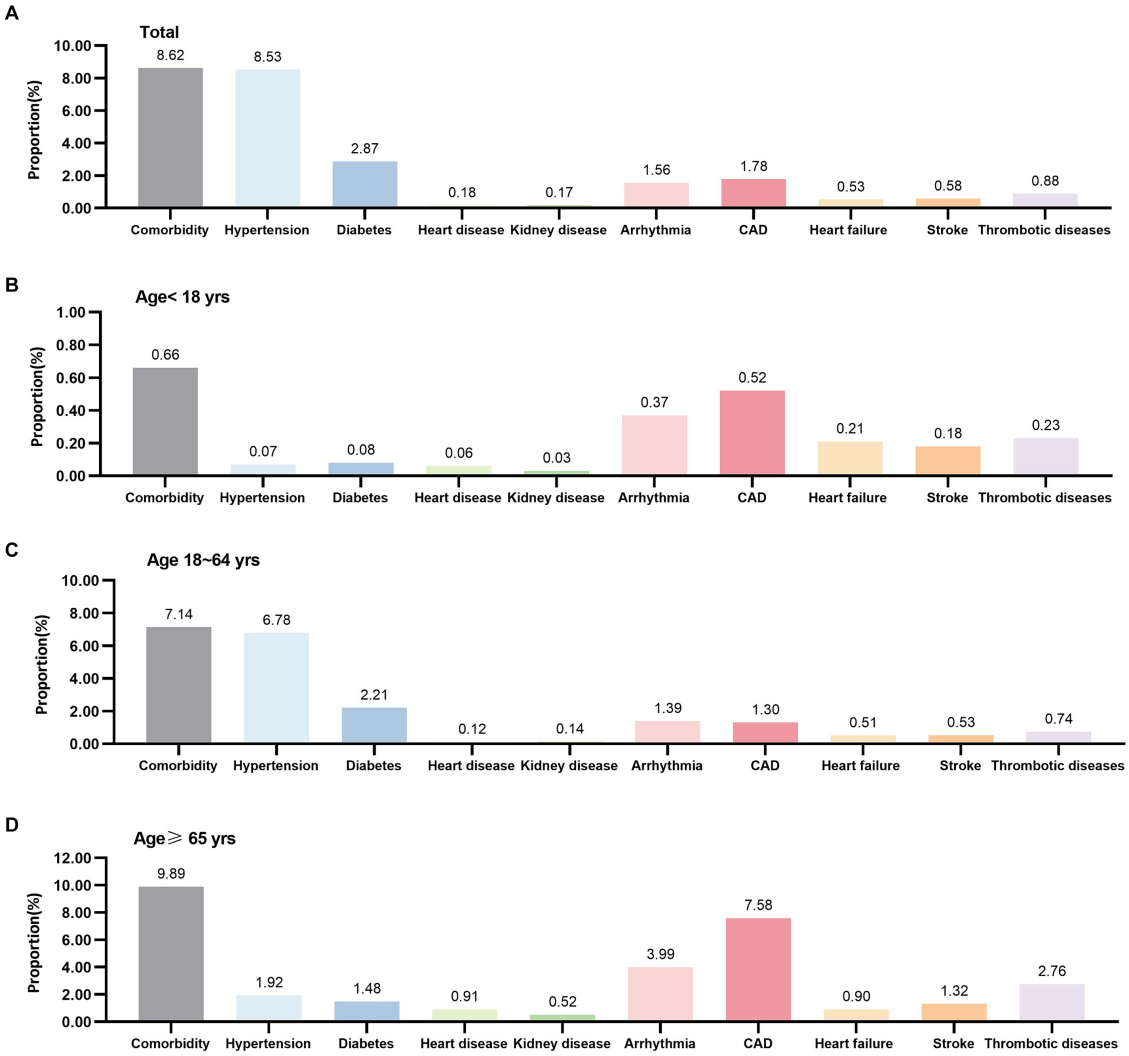
Comorbidity of COVID-19 patients in Fangcang shelter hospitals. CAD, coronary artery disease.

The characteristics of the participants from different age groups were significantly different. Those aged ≥65 years had the lowest proportion of male participants (52.77%), the highest proportion of participants with missing vaccinations (40.68%), were diagnosed with mild infection (27.76%), and had the longest duration of stay in the Fangcang shelter hospitals. In contrast, those aged<18 years had the lowest proportion of receiving three doses of the vaccine (1.84%) and had mild infection (21.29%) (Table 1). Differences between the age groups could also be observed regarding comorbidities, including hypertension, diabetes, heart disease, kidney disease, arrhythmia, coronary artery disease (CAD), heart failure, stroke, and thrombotic diseases (Figure 2).

### 3.2 Prevalence and correlates of insomnia

The prevalence of insomnia was 3.13% overall and increased by age groups (<18 years: 0.23%, as a reference in the regression model; 18–64 years: 2.64%, aOR = 10.11, 95% CI: 6.91–14.81; ≥65 years: 10.36%, aOR = 32.64, 95% CI: 22.23–47.92). Women and those who came from central districts had a significantly higher prevalence of insomnia. The prevalence of insomnia for the unvaccinated, one- or two-dose, and three-dose groups was 4.15, 2.71, and 2.95%, respectively, with an aOR of 0.85 (95% CI: 0.80–0.90) for the one- or two-dose group and an aOR of 0.84 (95% CI: 0.80–0.89) for the three-dose group. Patients with mild symptoms had a higher prevalence of insomnia, with an aOR of 1.09 (95% CI:1.04–1.15). Regarding the location of Fangcang shelter hospitals, patients in NECC had the highest prevalence of insomnia (3.63%). It was observed that an extra day in the hospital significantly increased the risk of insomnia by 12% (aOR = 1.12, 95% CI: 1.11–1.13, and *p* < 0.001) (Table 2).

**Table 2:** Risk factors of insomnia among COVID-19 patients.

Variable	Prevalence (cases/participants, ‰)	aOR	*p*-value	*p* for interaction with age groups
Total	3.13 (7,501/239,448)	—	—	—
*Age groups, years*				
<18	0.23 (26/11,305)	Reference		—
18–64	2.64 (5,522/209,299)	10.71 (6.63–17.3)	**<0.001**	—
≥65	10.36 (1,953/18,844)	30.36 (18.73–49.24)	**<0.001**	—
*Sex*				
Female	3.81 (3,727/97,956)	Reference		*p*_(18–64 years × male)_ = 0.090
Male	2.67 (3,774/141,492)	0.88 (0.83–0.94)	**<0.001**	*p*_(≥65 years × male)_ = 0.185
*Marital status*				
Unmarried	2.20 (1,940/88,154)	Reference		*p*_(18–64 years × married)_ < 0.001
Married	3.64 (5,219/143,409)	1.12 (1.05–1.20)	**<0.001**	*p*_(18–64 years × others)_ = 0.251
Others	4.34 (342/7,885)	1.22 (1.05–1.42)	**<0.001**	
*Number of vaccine doses*				
None	4.15 (2,250/54,169)	Reference		*p*_(18–64 years × 1–2 doses)_ = 0.024
One or two	2.71 (2,196/81,122)	0.91 (0.84–0.98)	**<0.001**	*p*_(≥65 years × 1 ~ 2 doses)_ = 0.034
				*p*_(18–64 years × 3 doses)_ = 0.148
Three	2.95 (2,975/100,748)	0.91 (0.85–0.98)	**<0.001**	*p*_(≥65 years × 3 doses)_ = 0.166
*Discharge diagnosis*				
Asymptomatic	2.86 (5,026/175,778)	Reference		*p*_(18–64 years × mild)_ = 0.080
Mild	3.89 (2,475/63,670)	1.09 (1.04–1.15)	**0.001**	*p*_(≥65 years × mild)_ = 0.006
*Location of Fangcang shelter hospitals*				
NECC	3.63 (6,077/167,367)	Reference		*p*_(18–64 years × WEM)_ = 0.699
WEM	1.70 (435/25,593)	0.05 (0.04–0.07)	**<0.001**	*p*_(18–64 years × SNIEC)_ = 0.334
				*p*_(18–64 years × WEM)_ *=* 0.496
SNIEC	2.13 (989/46,488)	0.39 (0.35–0.43)	**<0.001**	*p*_(≥65 years × SNIEC)_ = 0.021
*Place of residence*				
Central districts	4.25 (4,820/113,434)	Reference		*p*_(18–64 years × surrounding areas)_ = 0.974
Surrounding areas	2.13 (2,681/126,014)	0.56 (0.53–0.60)	**<0.001**	*p*_(≥65 years × surrounding areas)_ = 0.476
Length of hospital stay	—	1.12 (1.11–1.13)	**<0.001**	*p*_(18–64 years × length of hospital stay)_ = 0.602
				*p*_(≥65 years × length of hospital stay)_ = 0.662

aOR, adjusted odds ratio. Central districts in Shanghai included the Huangpu district, Xuhui district, Changning district, Yangpu district, Jing’an district, Hongkou district, and Putuo district. Surrounding areas indicated other districts excluding central districts. NECC, National Exhibition and Convention Center; WEM, World Expo Museum; SNIEC, Shanghai New International Expo Centre. Texts in bold type indicate statistical significance.

### 3.3 Age-specific prevalence and correlates of insomnia

The frequency of insomnia that increased with age groups could be observed among subgroups based on all demographic characteristics (all *p* < 0.001) (Table 3). When categorized by age groups, insomnia exhibited distinct patterns in different groups. No significant difference in the prevalence of insomnia was observed among participants with different sexes, marital status, number of vaccine doses, and residence places in the group that included participants aged <18 years. Participants with mild symptoms who stayed in the NECC had a higher prevalence of insomnia. A significantly lower prevalence of insomnia was observed in the group that included 18–64 years old, male, unmarried, vaccinated, and asymptomatic patients, and residents of surrounding areas. In the group that included patients aged ≥65 years, male patients, vaccinated patients, surrounding area residents, and those who were treated in the WEM, a significantly lower prevalence of insomnia was observed, while no significant difference was observed between asymptomatic patients and patients with mild symptoms (Table 3).

**Table 3:** Age-specific prevalence of insomnia among COVID-19 patients.

Variable	Age groups			*p*-value
	<18 years	18–64 years	≥65 years	
*Sex*				
Female	0.18 (9/4,903)	3.24 (2,728/81,416)	11.12 (990/7,910)	**<0.001**
Male	0.27 (17/6,376)	2.23 (2,794/122,361)	9.68 (963/8,981)	**<0.001**
*p*-value	0.363	**<0.001**	**0.001**	
*Marital status*				
Unmarried	0.23 (26/11,076)	2.16 (1,604/72,531)	10.63 (310/2,607)	**<0.001**
Married	0 (0/0)	2.89 (3,717/124,899)	10.15 (1,502/13,291)	**<0.001**
Others	0 (0/203)	3.07 (201/6,347)	12.43 (141/993)	**<0.001**
*p*-value	0.49	**<0.001**	**0.046**	
*Number of vaccine doses*				
None	0.11 (5/4,364)	3.23 (1,363/40,834)	11.6 (882/6,721)	**<0.001**
One or two	0.3 (20/6,677)	2.46 (1,711/67,794)	9.45 (465/4,455)	**<0.001**
Three	0.48 (1/206)	2.53 (2,385/91,987)	9.55 (589/5,580)	**<0.001**
*p*-value	0.106	**<0.001**	**<0.001**	
*Discharge diagnosis*				
Asymptomatic	0.16 (14/8,884)	2.35 (3,606/149,661)	10.33 (1,406/12,207)	**<0.001**
Mild	0.5 (12/2,395)	3.42 (1,916/54,116)	10.46 (547/4,684)	**<0.001**
*p*-value	**0.002**	**<0.001**	**0.795**	
*Location of Fangcang shelter hospitals*				
NECC	0.32 (23/7,077)	3.15 (4,623/142,095)	10.56 (1,431/12,118)	**<0.001**
WEM	0.09 (1/1,121)	1.52 (352/22,779)	6.12 (82/1,258)	**<0.001**
SNIEC	0.06 (2/3,081)	1.39 (547/38,903)	11.13 (440/3,515)	**<0.001**
*p*-value	**0.025**	**<0.001**	**<0.001**	
*Place of residence*				
Central districts	0.29 (17/5,756)	3.59 (3,447/92,554)	11.63 (1,356/10,304)	**<0.001**
Surrounding areas	0.16 (9/5,523)	1.83 (2,075/111,223)	8.31 (597/6,587)	**<0.001**
*p*-value	0.144	**<0.001**	**<0.001**	

Central districts in Shanghai included the Huangpu district, Xuhui district, Changning district, Yangpu district, Jing’an district, Hongkou district, and Putuo district. Surrounding areas indicated other districts excluding central districts. NECC, National Exhibition and Convention Center; WEM, World Expo Museum; SNIEC, Shanghai New International Expo Centre. Texts in bold type indicate statistical significance.

**Figure 3:**
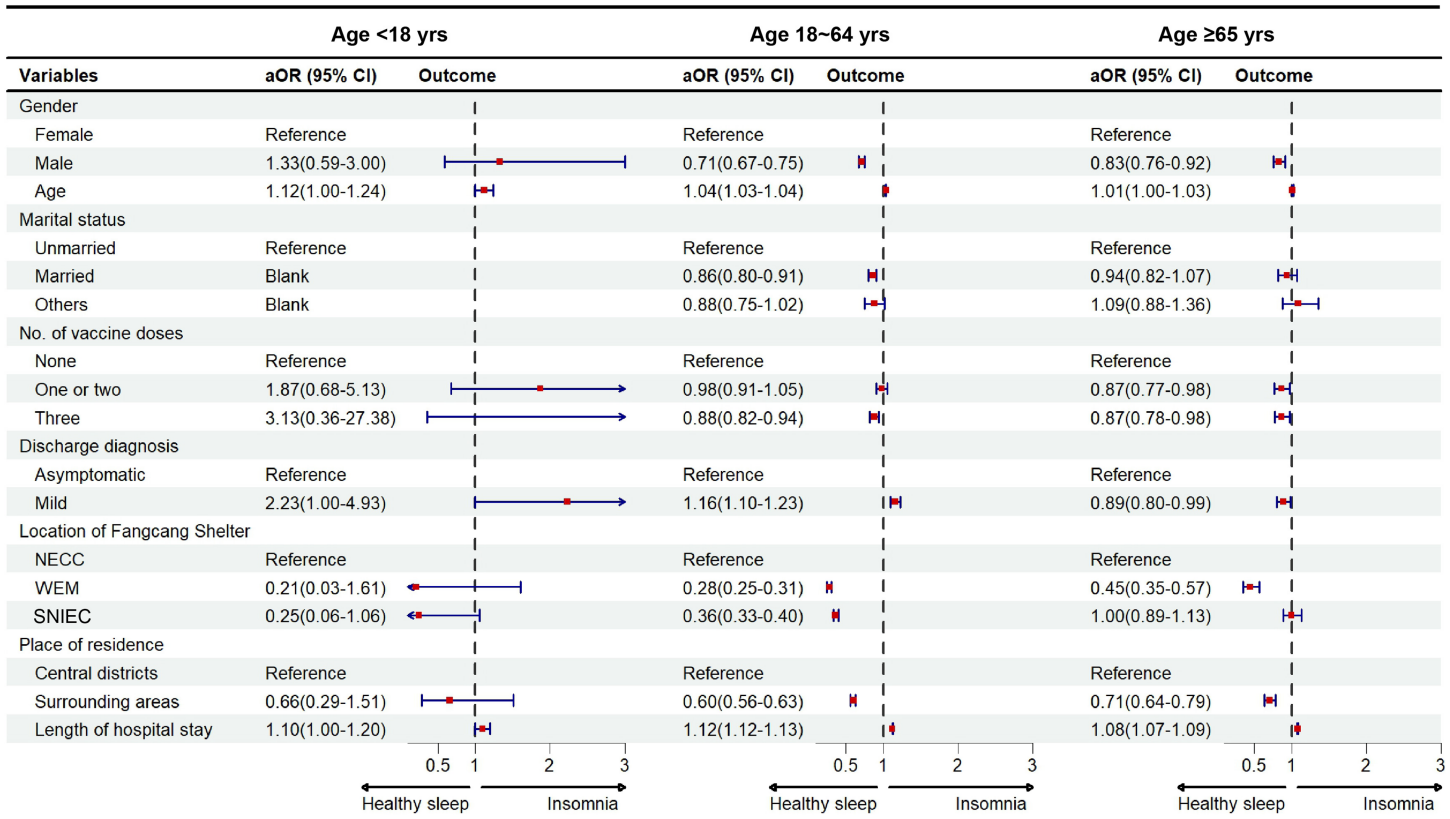
Risk factors of insomnia categorized by age groups among COVID-19 patients aOR, adjusted odds ratio. Central districts in Shanghai included the Huangpu district, Xuhui district, Changning district, Yangpu district, Jing’an district, Hongkou district, and Putuo district. Surrounding areas indicated other districts excluding central districts. NECC, National Exhibition and Convention Center; WEM, World Expo Museum; SNIEC, Shanghai New International Expo Centre.

Figure 3 shows the results of the multivariate regression analysis for the three age groups. In the group aged <18 years, age (aOR = 1.12, 95% CI: 1.00–1.24), mild symptoms (aOR = 2.23, 95% CI: 1.00–4.93), and a longer stay in the hospital (aOR = 1.10, 95% CI: 1.00–1.20 per day) were significantly associated with a higher risk of insomnia. In the group aged 18–64 years, being male (aOR = 0.71, 95% CI: 0.67–0.75), age (aOR = 1.04, 95% CI: 1.03–1.04), mild symptoms (aOR = 1.16, 95% CI: 1.10–1.23), and an extra day of staying in the hospital (aOR = 1.12, 95% CI: 1.12–1.13) were positively associated with insomnia. In addition to being treated in two other Fangcang shelter hospitals compared with the NECC, living in surrounding areas and vaccination were negatively associated with the risk of insomnia. In particular, only vaccination in three doses (aOR = 0.88, 95% CI: 0.82–0.94) had a significant association with insomnia. In the group aged ≥65 years, being male (aOR = 0.83, 95% CI: 0.76–0.92) and an extra day of staying in the hospital (aOR = 1.08, 95% CI: 1.07–1.09) were positively associated with a higher risk of insomnia. While vaccination, mild symptoms, being treated in the WEM, and living in surrounding areas were negatively associated with the risk of insomnia. One or two doses (aOR = 0.87, 95% CI: 0.77–0.98) and three doses (aOR = 0.87, 95% CI: 0.78–0.98) were significantly associated with a lower risk of insomnia (Figure 3).

## 4 Discussion

To the best of our knowledge, this is the first large-scale study that investigated the age-specific prevalence and correlates of insomnia among patients infected with SARS-COV-2, who were admitted to Fangcang shelter hospitals based on prescription drug records during the period of the Omicron wave from 26 March 2022 to 31 May 2022. We investigated 2,39,448 patients in three Fangcang shelter hospitals, namely the NECC, the WEM, and the SNIEC. Our results showed that the prevalence of insomnia increased with age and was 0.23, 2.64, 10.36, and 3.13% for the groups aged <18 years, 18–64 years, ≥65 years, and overall, respectively. Vaccination was found to be associated with a reduced risk of insomnia for adults, especially for those aged ≥65 years.

In Spain, 75.2% of the general population had sleep problems during the lockdown (20). In France, 56% of the population had poorer sleep quality, 48% had less regular sleep schedules, 30% slept less, and 33% slept more during the lockdown (21). Among COVID-19 inpatients in China, the prevalence of insomnia was 42.8% (22). Our study evaluated the prevalence of insomnia during the late period of the pandemic. Previous studies have investigated sleep problems using self-reported questionnaires (8, 20, 21, 23). According to the criteria of insomnia based on medication prescription in this study, the overall prevalence of insomnia during the late period of the pandemic was 3.1%.

In our study, the prevalence of insomnia was observed to be the highest in the group aged ≥65 years, with a nearly 31 times higher risk of insomnia than the group aged <18 years and a three times higher risk than the group aged 18–64 years. A recent study reported that in recent years, Americans who were 65 years of age or older experienced a larger increase in the prevalence of trouble sleeping than the other two younger age groups, whereas when compared to the middle-aged group (24), they experienced a similar prevalence of trouble sleeping. All these studies shared a common observation, which was that sleep problems were less frequent among young people. It has been acknowledged that older adults do not sleep as well as younger adults because changes occur as we grow older in both the macro-level architecture of sleep, such as sleep length and phases, and the micro-level architecture of sleep, such as the amount and quality of sleep oscillations (25). Against the background of the SARS-COV-2 pandemic, continuous bright light, even at night, and environmental noises may accelerate insomnia among older adults who are prone to sleep problems.

Our study reported that the SARS-COV-2 vaccine was significantly associated with the risk reduction of insomnia in adult patients. The homeostatic regulation of physiological sleep can be regulated by the immune system through the release of regulatory substances such as the cytokines tumor necrosis factor and interleukin caused by acute or chronic immune activation (26). A previous study showed that patients with mild symptoms had lower levels of inflammation and a higher level of neutralizing antibodies (27). Therefore, vaccination may make a significant contribution to sleep homeostasis in the context of infectious diseases. This might also explain the phenomenon that asymptomatic patients had a lower prevalence of insomnia because of their lower inflammation levels. Moreover, as stress and worries are considered important factors that disturb falling asleep or the quality of sleep (28), vaccinated patients may have fewer worries about their health status, and therefore, have fewer sleep problems. Receiving the first dose of the COVID-19 vaccine led to significant improvements in mental health among US adults. Another study conducted in the US also showed that COVID-19 vaccination was significantly associated with declines in the level of distress and perceived risks of infection among adults (29). Similarly, a short-term improvement in depressive and anxiety symptoms was observed among Swedish adults who received COVID-19 vaccination during the pandemic (30). In this study, 40.7% of people aged ≥65 years did not take the SARS-COV-2 vaccine, which may have partially accelerated their worries about their health status when staying in the open ward of Fangcang shelter hospitals (31). No significant association between vaccination and insomnia was observed among the participants of the group aged <18 years, which might be due to their immune system or psychological state. Interestingly, only three doses of vaccine had a significant association with a reduced risk of insomnia for the group aged 18–64 years. Contrarily, vaccination, regardless of the number of doses, decreased the risk of insomnia for the group aged ≥65 years. This result highlighted the importance and benefit of a vaccine promotion program for the older adult. Future research could further investigate whether vaccines have an influence on insomnia through some physiological mechanisms.

We found that women were more likely to have a higher risk of insomnia; this finding agreed with another study on Chinese inpatients (22). However, a previous study found no significant association between sex and insomnia among COVID-19 patients, who experienced sleep disturbances 2 months after discharge from the hospital (32). Another study on US adults suggested that the prevalence of trouble sleeping and prescription medications for insomnia was higher in women, while the prevalence of short sleep duration and sleep disorders was higher in men (33), which suggested a more complicated sex-specific difference regarding sleep problems. The increased prevalence of sleep disorders during the COVID-19 pandemic has also been highlighted, which may be due to isolation, quarantine, financial loss, and other psychosocial factors (34). Therefore, the result of our study might suggest that women were prone to infectious disease disasters and highlight the importance of psychological intervention for vulnerable populations. Other distinct age-specific correlates of insomnia were also observed in this study. In the middle-aged group, marriage was significantly associated with a lower risk of insomnia, while this correlation was not observed in the group aged ≥65 years. This might have been caused by other complex socio-psychological factors that might need to be further explored.

There are some limitations to this study. First, although the prevalence of insomnia was assessed using prescribed medication, it was not measured using ICSD-D and DSM-5 criteria, actigraphy and polysomnography, or any other sleep scales; it depended on the subjective medical needs of infected patients to some extent. Our study did not include the proportion of patients who had less severe insomnia and did not have medications. A stricter criterion for evaluating insomnia in this study could have led to a lower frequency of insomnia problems, as it has been previously reported that the number of people who experience trouble sleeping is far greater than that of those who use medications commonly prescribed for insomnia (33). However, electronic medical records are the most convenient and precise tools for such a large-scale study. We also showed the detailed prevalence of insomnia defined by two medications (Supplementary Table S4). Second, all patients included had mild symptoms or were asymptomatic patients who were infected during the period when Omicron was prevalent. Since severe respiratory diseases might affect sleep, the results generated from this study might not be generalizable to all populations. Third, we did not have records of previous insomnia history for patients in Fangcang shelter hospitals or the medical care service provided at different study periods; these need to be further explored. However, to avoid ascertainment bias as much as possible, we excluded patients with a self-reported history of psychiatric disorders. Finally, this was a cross-sectional study. Hence, it could not assess temporality or causation between factors of interest and the development of insomnia.

## 5 Conclusion

In conclusion, our findings suggested that the prevalence and risk factors of insomnia differed in different age groups, with older people being a high-risk population. Vaccination was beneficial to reduce the risk of insomnia in adults, especially the older adult. More attention should be paid to vulnerable subpopulations, such as women, unvaccinated individuals, those who are single, those residing in central districts, and those who stayed at Fangcang shelter hospitals for a long time. In addition, a comprehensive approach might strengthen interventions related to the mental health impact caused by the pandemic.

### What is already known on this topic

There is sufficient evidence for the occurrence of sleep problems among various subpopulations during the early stage of the COVID-19 pandemic. However, the evidence for sleep problems is based almost entirely on self-reported questionnaires. In addition, most epidemiological studies were also performed among patients infected with Alpha and Delta variants, both of which showed different features from the Omicron variant. Moreover, Fangcang shelter hospitals are characterized by the high volume of patient admissions, open wards, and adaption to the requirement of treatments. Patients admitted to Fangcang shelter hospitals might exhibit different characteristics in sleep problems. We updated the search using PubMed from 2019 until 31 October 2023, for the keywords “insomnia,” “COVID-19,” and “Fangcang shelter hospital” to specify the quarantine places where patients stayed and identified only seven relevant studies. Five of the seven studies were performed when the pandemic was dominated by Alpha and Delta variants, and the other two studies investigated sleep or mental health problems among SARS-CoV-2-infected patients using non-probability sampling. No age-specific issues were investigated in consideration of the age-dependent changes in sleep. In summary, the prevalence and risk factors of insomnia (e.g., the effect of vaccination, the size of Fangcang shelter hospitals, and the length of hospital stay) among different age groups when quarantined in Fangcang shelter hospitals during the late period of the pandemic remains unclear.

### What this study adds

This large-scale study is the first to investigate the age-specific prevalence of insomnia among Omicron-infected patients admitted to Fangcang shelter hospitals. Based on prescription medication records, we investigated the prevalence of insomnia among Omicron-infected patients, most of whom were asymptomatic. The prevalence of insomnia was relatively low compared to the study conducted in the pre-Omicron period, and insomnia was most prevalent among patients ≥65 years. This study also provided evidence for the effect of vaccination on insomnia: in an age-specific regression model, three doses of vaccine were observed to be significantly associated with a reduced risk of insomnia for patients aged 18–64 years, while vaccination regardless of the number of doses was significantly associated with a reduced risk of insomnia for patients aged ≥65 years. Moreover, patients admitted to smaller Fangcang shelter hospitals had a lower prevalence of insomnia, and an extra day in the hospital significantly increased the risk of insomnia for all age groups.

### How this study might affect research, practice, or policy

During the period of the Omicron wave, all individuals infected with the Omicron variant were admitted to Fangcang shelter hospitals. This epidemiological study provided evidence for the age-specific prevalence and risk factors of insomnia among patients with mild symptoms and asymptomatic SARS-CoV-2 infected patients in Fangcang shelter hospitals. With the older adult being a high-risk population for insomnia, they would benefit from the intervention of a SARS-CoV-2 vaccine program for both physical and physiological reasons. Individuals belonging to different age groups are prone to insomnia due to various factors, which highlights the importance of age-oriented treatment and care. Intervention on the delayed impact of the pandemic on insomnia holds great potential to support both sleep health goals and combat infectious diseases. This finding is especially important in terms of interventions for sleep problems in situations where infectious diseases break out and a large number of patients gather in places such as Fangcang shelter hospitals..

## Data availability statement

The original contributions presented in the study are included in the article/Supplementary material, further inquiries can be directed to the corresponding authors.

## Ethics statement

The studies involving humans were approved by the Institutional Review Board (IRB) of Ruijin Hospital affiliated to Shanghai Jiao Tong University School of Medicine. The studies were conducted in accordance with the local legislation and institutional requirements. Written informed consent for participation in this study was provided by the participants’ legal guardians/next of kin.

## Author contributions

RS: Conceptualization, Data curation, Formal Analysis, Funding acquisition, Investigation, Methodology, Writing – original draft, Writing – review & editing. YW: Conceptualization, Investigation, Resources, Writing – review & editing, Data curation. YC: Investigation, Methodology, Resources, Writing – review & editing. ZY: Investigation, Project administration, Writing – review & editing. FJ: Project administration, Supervision, Writing – review & editing. HS: Project administration, Writing – review & editing. EC: Funding acquisition, Project administration, Resources, Supervision, Validation, Writing – review & editing. YZ: Conceptualization, Data curation, Funding acquisition, Methodology, Supervision, Writing – review & editing.
