# Supplementary material for: Age-specific disparity in insomnia among COVID-19 patients in Fangcang shelter hospitals: a population-based study in Shanghai, China
Source: Front Neurol. 2024 Jul 26;15:1420898. doi: 10.3389/fneur.2024.1420898 (PMC11310121; doi:10.3389/fneur.2024.1420898)
Supplement: Supplementary file 1 [file Data_Sheet_1.docx]

**Table S1 Characteristics of three Fangcang shelter hospitals**

| **Items** | **National Exhibition and Convention Center** | **New International Expo Center** | **World Expo Museum** |
| --- | --- | --- | --- |
| Areas | About 600,000 square meters | More than 300,000 square meters | About 21,900 square meters |
| No. of beds | About 50,000 | More than 15,000 | About 7300 |
| Location | Qingpu district | Pudong district | Huangpu district |
| Open date | 26^th^ March 2022 | 9^th^ April 2022 | 26^th^ March 2022 |
| Close-down date | 31^st^ May 2022 | 31^st^ May 2022 | 25^th^ May 2022 |
| No. of patients | 169922 | 46695 | 25647 |
| Open days | 67 | 53 | 67 |
| Daily patients | 2536 | 881 | 382 |

**Table S2 Psychiatric medication used among COVID-19 patients in Fangcang shelters**

| **Psychiatric medication** | **Number of cases** | **Pharmaceutical uses** |
| --- | --- | --- |
| Zolpidem | 4796 | Insomnia |
| Estazolam | 3381 | Anxiety or sleep disorder |
| Clonazepam | 18 | Epilepsy, anxiety or sleep disorder |
| Risperidone | 51 | Schizophrenia |
| Olanzapine | 195 | Schizophrenia |
| Quetiapine | 4 | Schizophrenia |
| Escitalopram | 14 | Depression |
| Paroxetine | 34 | Depression |
| Sertraline | 41 | Depression |
| Venlafaxine | 23 | Depression |
| Flupentixol-melitracen | 101 | Depression |

**Table S3 Characteristics of COVID-19 patients by prescription medication**

| Variable | None | Zolpidem | Estazolam | Both | *P* value |
| --- | --- | --- | --- | --- | --- |
| Total, n | 231,947 | 4,120 | 2,705 | 676 |  |
| Age, median (IQR) | 41(30-54) | 53(37-63) | 58(47-65) | 63(55-68) | **<0.001** |
| Sex |  |  |  |  | **<0.001** |
| Female | 94229(40.63) | 1787(43.37) | 1575(58.23) | 365(53.99) |  |
| Male | 137718(59.37) | 2333(56.63) | 1130(41.77) | 311(46.01) |  |
| Marital status |  |  |  |  | **<0.001** |
| Unmarried | 86214(37.17) | 1215(29.49) | 596(22.03) | 129(19.08) |  |
| Married | 138190(59.58) | 2718(65.97) | 1984(73.35) | 517(76.48) |  |
| Others | 7543(3.25) | 187(4.54) | 125(4.62) | 30(4.44) |  |
| Place of residence |  |  |  |  | **<0.001** |
| Central districts | 108614(46.83) | 2560(62.14) | 1743(64.44) | 517(76.48) |  |
| Surrounding areas | 123333(53.17) | 1560(37.86) | 962(35.56) | 159(23.52) |  |
| Numbers of vaccine doses |  |  |  |  | **<0.001** |
| None | 51919(22.71) | 1093(26.84) | 889(33.21) | 268(39.88) |  |
| One or two | 78926(34.52) | 1252(30.75) | 762(28.46) | 182(27.08) |  |
| Three | 97773(42.77) | 1727(42.41) | 1026(38.33) | 222(33.04) |  |
| Discharge diagnosis |  |  |  |  | **<0.001** |
| Mild | 170752(73.62) | 2938(71.31) | 1678(62.03) | 410(60.65) |  |
| Asymptomatic | 61195(26.38) | 1182(28.69) | 1027(37.97) | 266(39.35) |  |
| Location of Fangcang shelter |  |  |  |  | **<0.001** |
| NECC | 161290(69.54) | 3646(88.5) | 1845(68.21) | 586(86.69) |  |
| WEM | 25158(10.85) | 44(1.07) | 387(14.31) | 4(0.59) |  |
| NIEC | 45499(19.62) | 430(10.44) | 473(17.49) | 86(12.72) |  |
| Days of hospital stay, median (IQR) | 7.0(5.0-10.0) | 8.0(6.0-11.0) | 9.7(6.5-12.8) | 11.0(8.0-14.0) | **<0.001** |

Note: Central districts in Shanghai including Huangpu district, Xuhui district, Changning district, Yangpu district, Jing’an district, Hongkou district, Putuo district. Surrounding areas indicated other districts except central districts. NECC, the National Exhibition and Convention Center; WEM, World Expo Museum; NIEC, New International Expo Center.

**Table S4 Prevalence of insomnia by two respective medications**

| Variable | Prevalence by Zolpidem (Cases/participants, ％) | Prevalence by Estazolam (Cases/participants，％) |
| --- | --- | --- |
| Total | 2.00 (4796/239448) | 1.41 (3381/239448) |
| Age groups, yrs |  |  |
| <18 | 1.50 (17/11305) | 0.80 (9/11305) |
| 18-64 | 1.73 (3618/209299) | 1.10 (2294/209299) |
| ≥65 | 6.16 (1161/18844) | 5.72 (1078/18844) |
| Sex |  |  |
| Female | 2.20 (2152/97956) | 1.98 (1940/97956) |
| Male | 1.87 (2644/141492) | 1.02 (1441/141492) |
| Marital status |  |  |
| Unmarried | 1.53 (1344/88154) | 0.82 (725/88154) |
| Married | 2.26 (3235/143409) | 1.74 (2501/143409) |
| Others | 2.75 (217/7885) | 1.97 (155/7885) |
| Numbers of vaccine doses |  |  |
| None | 2.51 (1361/54169) | 2.14 (1157/54169) |
| One or two | 1.77 (1434/81122) | 1.16 (944/81122) |
| Three | 1.94 (1949/100748) | 1.24 (1248/100748) |
| Discharge diagnosis |  |  |
| Asymptomatic | 1.91 (3348/175778) | 1.19 (2088/175778) |
| Mild | 2.22 (1448/63670) | 2.03 (1293/63670) |
| Location of Fangcang shelter |  |  |
| NECC | 2.53 (4232/167367) | 1.45 (2431/167367) |
| WEM | 0.19 (48/25593) | 1.53 (391/25593) |
| NIEC | 1.11 (516/46488) | 1.20 (559/46488) |
| Place of residence |  |  |
| Central districts | 2.71 (3077/113434) | 1.99 (2260/113434) |
| Surrounding areas | 1.36 (1719/126014) | 0.89 (1121/126014) |
| Length of hospital stay | - |  |

Note: Central districts in Shanghai including Huangpu district, Xuhui district, Changning district, Yangpu district, Jing’an district, Hongkou district, Putuo district. Surrounding areas indicated other districts except central districts. NECC, the National Exhibition and Convention Center; WEM, World Expo Museum; NIEC, New International Expo Center.

**
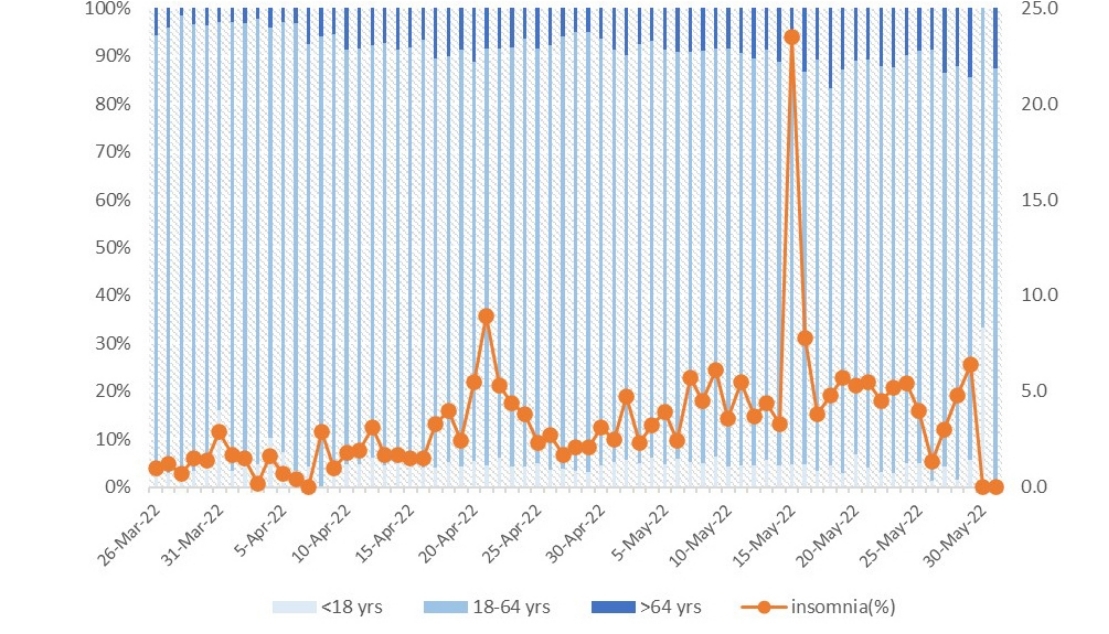
**

**Figure S1 prevalence of insomnia by admission date in Fangcang shelter hospitals**


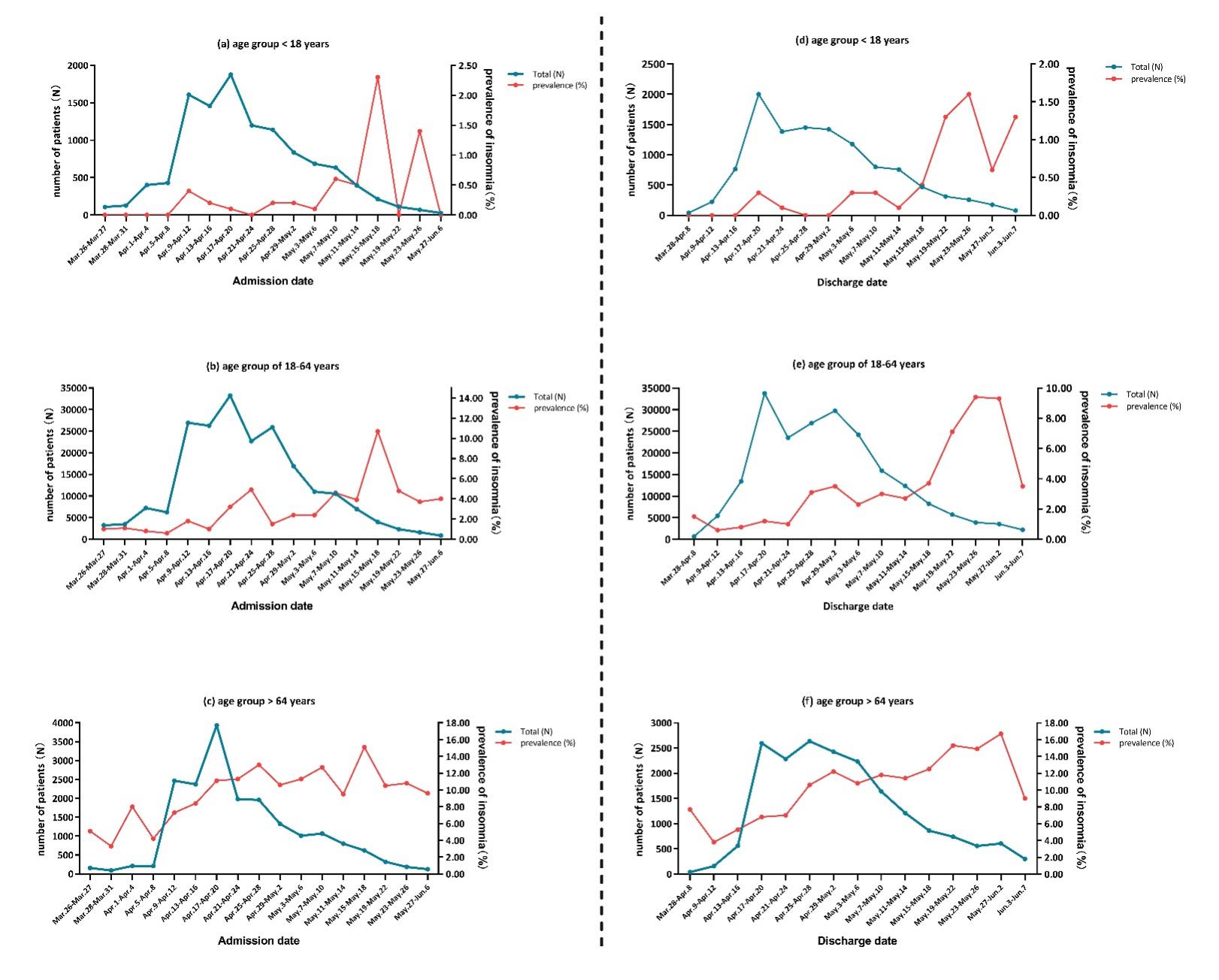
**Figure S2 The number of patients and prevalence of insomnia by admission and discharge date**
